# Comparison of phylogenetic trees defined on different but mutually overlapping sets of taxa: A review

**DOI:** 10.1002/ece3.70054

**Published:** 2024-08-08

**Authors:** Wanlin Li, Aleksandr Koshkarov, Nadia Tahiri

**Affiliations:** ^1^ Department of Computer Science University of Sherbrooke Sherbrooke Quebec Canada

**Keywords:** consensus tree, phylogenetic tree, supertree, tree distance

## Abstract

Phylogenetic trees represent the evolutionary relationships and ancestry of various species or groups of organisms. Comparing these trees by measuring the distance between them is essential for applications such as tree clustering and the Tree of Life project. Many distance metrics for phylogenetic trees focus on trees defined on the same set of taxa. However, some problems require calculating distances between trees with different but overlapping sets of taxa. This study reviews state‐of‐the‐art distance measures for such trees, covering six major approaches, including the constraint‐based Robinson–Foulds (RF) distance RF(−), the completion‐based RF(+), the generalized RF (GRF), the dissimilarity measure, the vectorial tree distance, and the geodesic distance in the extended Billera‐Holmes‐Vogtmann tree space. Among these, three RF‐based methods, RF(−), RF(+), and GRF, were examined in detail on generated clusters of phylogenetic trees defined on different but mutually overlapping sets of taxa. Additionally, we reviewed nine related techniques, including leaf imputation methods, the tree edit distance, and visual comparison. A comparison of the related distance measures, highlighting their principal advantages and shortcomings, is provided. This review offers valuable insights into their applicability and performance, guiding the appropriate use of these metrics based on tree type (rooted or unrooted) and information type (topological or branch lengths).

## Graphical abstract

We explore six approaches, including Robinson–Foulds‐based distances, geodesic in the BHV tree space, and the dissimilarity measure, offering a comprehensive analysis. Our study compares these methods, discussing advantages, shortcomings, and practical insights into their applicability. The article provides a valuable contribution to the field, addressing the need for distance measures tailored to trees with distinct but overlapping taxa.

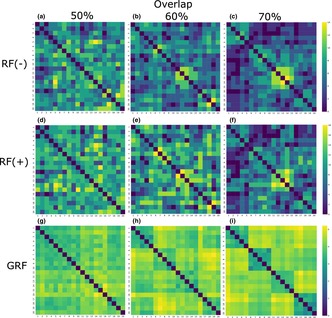

## 1 INTRODUCTION

Phylogenetic trees are essential to understanding evolution as they depict the relationships between genes, which are labeled on the leaves of the tree, and represent plausible scenarios of evolutionary events leading to the observed gene family from a common ancestral gene. These gene trees are usually derived from gene sequence alignments.

A phylogenetic tree is a type of leaf‐labeled tree where each inner node represents an ancestor of some species (i.e., taxa) with at least two children, and all leaves represent current species with different labels. The comparison of phylogenetic trees in terms of the distance between them is a promising task due to their practical relevance in tasks that include tree clustering and the Tree of Life construction.

Several methods have been developed to compare phylogenetic trees, utilizing various measures of distance (or similarity). The Robinson–Foulds distance (Robinson & Foulds, 1981) is a widely used metric that compares the topologies of two trees by counting the number of splits (bipartitions) that differ between them. The triplet and quartet distances (Critchlow et al., 1996; Estabrook et al., 1985) evaluate trees based on the consistency of relationships among groups of three or four taxa. The geodesic distance (Kupczok et al., 2008) measures the shortest path between two trees in a continuous tree space, capturing both topological and branch length differences.

The path distance (Steel & Penny, 1993) focuses on the differences in the paths between pairs of leaves in the two trees. The nodal distance (Cardona et al., 2010) assesses the dissimilarity based on the positions of internal nodes relative to the leaves. Maximum agreement subtrees (Amir & Keselman, 1997) identify the largest common subtree structure shared by two trees.

The Weighted Robinson–Foulds distance (wRF) (Robinson & Foulds, 1979) extends the Robinson–Foulds metric by incorporating branch length information, providing a more nuanced comparison. Kuhner and Felsenstein (1994) highlighted the importance of considering both topological and branch length‐based distance measures through their simulation comparisons of phylogeny algorithms under varying evolutionary rates. Kendall and Colijn (2016) introduced methods to map phylogenetic trees, revealing distinct evolutionary patterns through these mappings.

Other notable measures include the nearest‐neighbor interchange (NNI) distance (Li et al., 1996; Robinson, 1971) counts the minimum number of NNI operations needed to transform one tree into another. The subtree prune and regraft (SPR) distance (Hein, 1990) measures the minimum number of SPR operations required to convert one tree into another. The tree bisection and reconnection (TBR) distance (Allen & Steel, 2001) extends the SPR concept by allowing reconnections to any branch in the tree, providing a more flexible and comprehensive comparison method. The cophenetic correlation compares the pairwise distances between all pairs of taxa, incorporating both topological and branch length information (Sokal & Rohlf, 1962). The matching distance (Bogdanowicz & Giaro, 2013) considers the number of matched pairs of edges between trees, using topological information.

In practice, therefore, these distance measures assume that the two trees being compared have the same sets of leaves, which is often not the case. Moreover, some applied problems, such as supertree construction (Bansal et al., 2010; Tahiri et al., 2022; Whidden et al., 2014), phylogeny clustering (Silva & Wilkinson, 2021; Tahiri et al., 2018, 2022), tree of life construction (Hinchliff et al., 2015), and phylogenetic database searching (Chen et al., 2008; Wang et al., 2005) demand computing distances between trees with different but overlapping sets of taxa.

Gene trees are employed for the comparative analysis of evolutionary relationships among diverse species. To derive a dependable phylogeny for species, it is imperative to integrate gene trees with relevant information while mitigating topological conflicts (Maddison et al., 2007). This merging process is applicable to trees constructed for the same set of taxa, known as the consensus tree problem, or for different yet overlapping sets of taxa, termed the supertree problem (Sanderson et al., 1998).

The meticulous comparison and merging of trees, whether sharing identical or distinct but overlapping sets of leaves, represent crucial steps in the investigation of evolutionary relationships and the comprehensive understanding of species history.

This review focuses on distance measures for phylogenetic trees defined on different but mutually overlapping sets of taxa. We will start with approaches based on splits or bipartitions, which include metrics related to the popular Robinson–Foulds (RF) tree distance and the dissimilarity measure. These methods primarily focus on topological information and can be applied to both rooted and unrooted trees. Next, we will consider vector‐based distance measures, including the vectorial tree distance (VTD), which focuses on tree structure and hierarchy but disregards leaf names, and the geodesic in the extended Billera‐Holmes‐Vogtmann (BHV) tree space, which incorporates both topology and branch lengths. The VTD method is designed for rooted trees, while the BHV approach is typically used for unrooted trees but can also be adapted for rooted trees. Finally, we will mention several additional approaches that may be useful in the considered domain, such as leaf imputation techniques, the tree edit distance approach, and visual comparisons. All major distance measures will be compared, and a detailed comparison of the RF‐based metrics using a generated dataset of clusters of phylogenetic trees defined on different but overlapping sets of taxa will be provided. Throughout the review, we will provide guidance on the appropriate use of these distance measures depending on the type of trees (rooted or unrooted) and the type of information (topological or branch lengths) they utilize.

## 2 PRELIMINARIES AND NOTATIONS

Given a phylogenetic tree T, we denote by $V\left(T\right)$ its node set, $E\left(T\right)$ its edge set, and $L\left(T\right)$ its leaf set (i.e., taxa) with $L\left(T\right)\subset V\left(T\right)$. Let $C\left(T\right)$ be its set of clusters, where each cluster is a set of leaf labels for the descendants of a node in the tree. The subtree of T induced by the leaf set $L_{x}\left(T\right)$ is denoted as ${\left.T\right|}_{L_{x}\left(T\right)}$, a subset of $L\left(T\right)$. For any sets a and b, let a⊕b represent their symmetric difference, given by $a⊕b=\left(a\backslash b\right)\cup \left(b\backslash a\right)$, and its cardinality as ∣a⊕b∣=∣a∣+∣b∣−2∣a∩b∣.

In order to provide a comprehensive understanding of the methods and metrics used in our study, it is essential to define the concept of bootstrapping in the context of phylogenetic trees. *Bootstrapping* is a statistical method used in phylogenetic analysis to assess the reliability of the inferred trees (Felsenstein, 1985). In this context, bootstrap values are generated by repeatedly resampling the dataset and reconstructing the phylogenetic tree for each sample. The frequency with which a particular clade appears in these resampled trees is then calculated and expressed as a bootstrap percentage.

## 3 RELATED WORK

### 3.1 The constraint‐based RF(−)

One popular approach to compare two phylogenetic trees with only partially overlapping leaf sets is to first constrain (i.e., prune) both trees to their common leaf set. This method allows for the direct comparison of the resulting pruned trees using various distance measures. This approach has been effectively employed in several studies to facilitate tree comparison and analysis (Bansal, 2020; Bansal et al., 2010; Cotton & Wilkinson, 2007; Page, 2002).

The RF(−) approach can be expressed by the following equation:
(1)

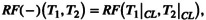

where CL is the common leaf set between $T_{1}$ and $T_{2}$, and ${\left.T\right|}_{\mathrm{CL}}$ denotes the subtree of T induced by CL.

**FIGURE 1:**
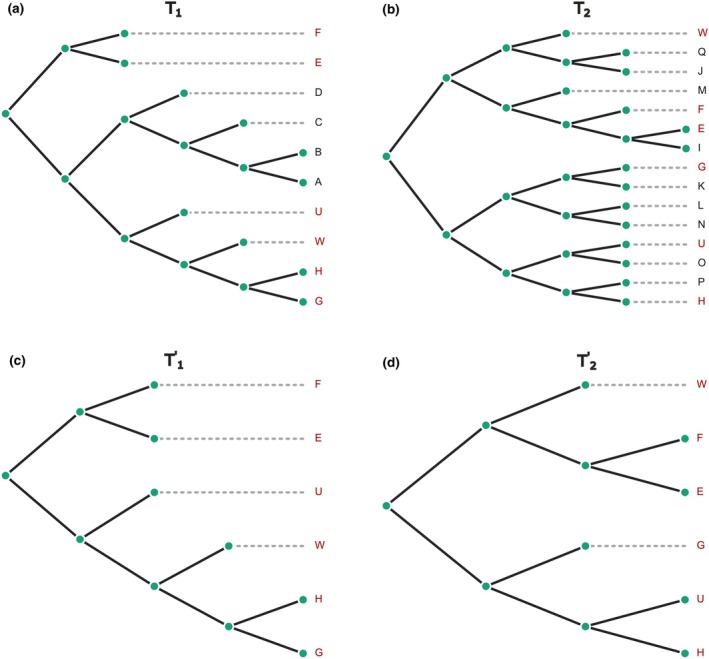
Tree Pruning. Panels (a and b) represent binary rooted trees $T_{1}$ and $T_{2}$ before pruning, respectively; shared taxa are highlighted in red. Panels (c and d) illustrate pruned binary rooted trees $T_{1}^{\prime }$ and $T_{2}^{\prime }$ after removing non‐shared taxa. The Robinson–Foulds (RF) distance between these two pruned trees is 4.

Figure 1 illustrates the constraint‐based RF(−) approach applied to two phylogenetic trees, $T_{1}$ and $T_{2}$. Tree $T_{1}$ has 10 leaves, while $T_{2}$ includes 15 leaves. The intersection of the two trees comprises 6 shared leaves highlighted in red, and the distinct leaves are marked in black. Trees $T_{1}^{\prime }$ and $T_{2}^{\prime }$ represent the pruned versions of $T_{1}$ and $T_{2}$, respectively, containing only the shared leaves.

The pruning process involves several steps. First, the set of leaves common to both trees $T_{1}$ and $T_{2}$ is identified. Next, the distinct leaves that are not part of the common leaf set are removed from each tree, which involves cutting off branches containing these distinct leaves. After removing the distinct leaves, the tree structure is adjusted to ensure it remains a valid binary tree, which may involve collapsing nodes that become redundant. The resulting trees, $T_{1}^{\prime }$ and $T_{2}^{\prime }$, are the pruned versions of $T_{1}$ and $T_{2}$, respectively, containing only the common leaves and retaining the original topological relationships among these leaves.

Trees $T_{1}^{\prime }$ and $T_{2}^{\prime }$ can then be compared using the RF(−) distance. In particular, the RF(−) distance between $T_{1}^{\prime }$ and $T_{2}^{\prime }$ is calculated as the classical RF distance for trees defined on the same set of taxa. In this example, $\mathrm{RF}\left(-\right)\left(T_{1}^{\prime },T_{2}^{\prime }\right)=4$.

Pruning the trees to include only the common taxa simplifies the comparison process, allowing for a straightforward calculation of the RF distance. This approach highlights the differences in the tree topologies that are directly related to the shared leaves. RF(−) can be applied to both rooted and unrooted trees, similar to the classic RF distance. However, this constraint‐based approach, while easy to understand and calculate, can lead to the loss of some valuable topological information by discarding leaves that are not shared by both trees. This is a major drawback of this method.

### 3.2 The completion‐based RF(+)

Another method for comparing trees with different leaf sets is to fill or complete each of the input trees with the union of their leaf sets in such a way as to minimize the RF distance between them. Although this approach is more sophisticated, it effectively accounts for all of the topological information from both trees being compared. In addition, the completion approach has the advantage of using a wider range of possible values, since it compares larger rather than smaller trees. In the case of using the RF distance in the tree completion process, the final distance measure is denoted as RF(+).

The idea of tree completion by means of the RF distance has been the subject of work by Cotton and Wilkinson (2007), where the authors introduced majority‐rule(+) supertrees, based on RF(+). This approach was later developed by Dong and Fernández‐Baca (2009) and Kupczok (2011). The concept of optimal tree completion has been investigated from various angles, considering different scenarios and approaches. The basic scenario, in which the taxa of one tree are a subset of the taxa of another tree, was discussed by Christensen et al. (2018). The authors introduced an algorithm called OCTAL (Optimal Completion of incomplete gene Trees ALgorithm), which can be used to seek the optimal solution for this case of nested trees using the Robinson–Foulds distance with a time complexity of $O\left(n^{2}\right)$, where n represents the total number of species. A further development of this approach was presented by Bansal (2020) with an implementation of this algorithm with complexity $O\left(n\right)$. More research on this problem has been conducted, including the restricted case in which the set of leaves of one tree is a subset of the set of leaves of another tree, and the more advanced case where the two trees have different numbers of overlapping leaves. This study was performed by Yao and Bansal (2021) in order to present the RF‐completion approach.

The calculation of RF(+) is based on the One Tree RF(+) problem (Bansal, 2020) and the Extraneous‐Clade‐Free RF(+) problem (Yao & Bansal, 2021). The constrained case with nested trees, called the One Tree RF(+) (OT‐RF(+)) problem (Bansal, 2020) for two trees $T_{1}$ and $T_{2}$, is described in terms of node colors. The algorithm assigns a green color to each node in $T_{1}$ if all the leaves within the subtree, with this node as its root, are present in both $T_{1}$ and $T_{2}$, a red color if all leaves of that subtree are present only in $T_{1}$, and a blue color if this subtree contains both green and red descendants. The algorithm additionally flags blue nodes that contain exactly one red child as “marked”. Then the least common ancestor (LCA) mapping defined on $L\left(T_{1}\left(v\right)\right)\cap L\left(T_{2}\right)$ for each blue or green node v of $T_{1}$ is calculated. The procedure continues with a pre‐order traversal of $T_{1}$, adding replicas of the red subtrees (containing only red nodes) in each marked node to the corresponding edges of $T_{2}$. The result is a tree $T^{\prime }$, which represents the optimal completion of $T_{2}$ on $L\left(T_{1}\right)$. The coloring scheme allows to distinguish leaves or subtrees that should be added to another tree. Furthermore, the authors show that there are optimal completions of $T_{1}$ and $T_{2}$, in which all added subtrees are maximal red or green subtrees. An optimal $O\left(n\right)$‐time algorithm for the OT‐RF(+) problem was presented in (Bansal, 2020; Yao & Bansal, 2021).

The restricted formulation of the RF(+) problem, called the Extraneous‐Clade‐Free RF(+) (EF‐RF(+)) problem, was proposed by Yao and Bansal (2021). This approach is based on a procedure that prevents the production of so‐called “extraneous” clades, that is, clades that consist of distinct leaves from both trees and do not contain leaves common to both trees. In the case of EF‐RF(+) for two trees $T_{1}$ and $T_{2}$, the completed trees $T_{1}^{\prime }$ and $T_{2}^{\prime }$ do not include any extraneous clades, and $\mathrm{RF}\left(T_{1}^{\prime },T_{2}^{\prime }\right)$ is minimized. The algorithm for this problem is based on the algorithm for the OT‐RF(+) problem, that is, first complete $T_{1}$ on $L\left(T_{1}\right)\cup L\left(T_{2}\right)$ relative to $T_{2}$ and then complete $T_{2}$ relative to the previous completion of $T_{1}$. The EF‐RF(+) problem for rooted trees can be solved in $O\left(n\right)$.

The exact RF(+) algorithm depends on the OT‐RF(+) and EF‐RF(+) problems for its core methodologies of tree completion, node coloring, and avoidance of extraneous clades, combining these elements to create an efficient and accurate distance measure for phylogenetic trees with overlapping taxa. In particular, the RF(+) method integrates the node coloring and LCA mapping techniques from the OT‐RF(+) problem to systematically complete the trees. By using these methods, the RF(+) algorithm can accurately add the necessary subtrees to create binary completions of the input trees. Furthermore, RF(+) leverages the EF‐RF(+) approach to avoid extraneous clades and iteratively complete the trees relative to each other. This ensures that the final completed trees are both optimal and free from unnecessary clades.

Yao and Bansal (2021) obtained several important results. First, the EF‐RF(+) distance between two trees is a 2‐approximation for the RF(+) distance between them and can serve as a heuristic for estimating RF(+) distances for large trees. Second, the exact algorithm they developed for the RF(+) problem has a complexity of $O\left({\mathrm{nk}}^{2}\right)$, where k is the number of maximal subtrees unique to an input tree. The value of k is bounded above by n, the total number of nodes in the tree. Third, extensive experiments showed that the RF(+) distance can yield different phylogenetic similarity inferences compared to the RF(−) distance. Finally, RF(+) employs only topological information about the phylogenetic trees being compared and can be applied to both rooted and unrooted trees.

**FIGURE 2:**
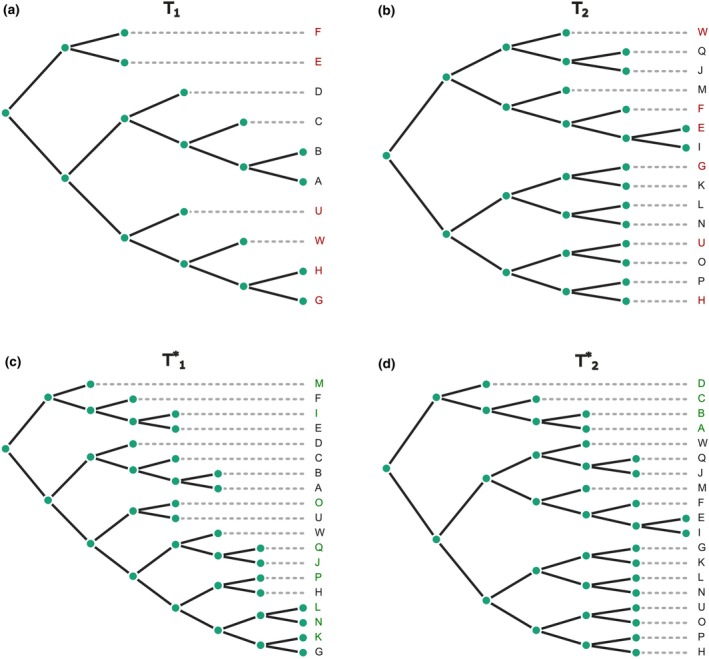
Illustration of tree completion for (a) $T_{1}$ and (b) $T_{2}$ prior to the completion step. (c) $T_{1}^{*}$ represents the binary completion of $T_{1}$, and (d) $T_{2}^{*}$ represents the binary completion of $T_{2}$. These completions are conducted on a union of trees in such a way that RF($T_{1}^{*}$, $T_{2}^{*}$) is minimized. Leaves in red color for $T_{1}$ and $T_{2}$ indicate shared taxa, while green leaves in $T_{1}^{*}$ and $T_{2}^{*}$ signify newly added leaves. The RF(+) distance in this example is equal to 6.

An example of the completion‐based RF(+) distance is shown in Figure 2. Tree completion results based on RF distance for trees from this example are as follows. The number of leaves in $T_{1}$ is 10, the number of leaves in $T_{2}$ is 15, the union of leaf sets is 19, the intersection of leaf sets is 6, and the RF(+) distance is 6. From the previous example, the result for RF(−) for the same pair of trees was 4.

### 3.3 The generalized Robinson–Foulds distance

In recent years, several generalizations of the RF distance have been suggested to deal with its drawbacks. Despite its widespread use over the decades, the RF distance has significant limitations (Llabrés et al., 2021). One major issue is its low resolution, as it can only produce a limited range of values based on the total number of leaves in the compared trees. This measure only checks if bipartitions are identical, treating small differences the same as large differences. Additionally, it can produce misleading results when comparing trees with different but overlapping sets of taxa, as it only considers topological differences. Llabrés et al. (2021) proposed a generalization of the RF distance that can be applied to phylogenetic trees for the same taxa or defined on different but overlapping set of taxa.

For phylogenetic trees $T_{1}$ and $T_{2}$ defined on the same or different but overlapped set of taxa, the equation for the Generalized Robinson–Foulds (GRF) distance is as follows:

(2) $$\begin{array}{c} \mathrm{GRF}\left(T_{1},T_{2}\right)=\frac{\sum_{a\in C\left(T_{1}\right)}\sum_{b\in C\left(T_{2}\right)\backslash C\left(T_{1}\right)}|a⊕b|}{|C\left(T_{1}\right)\cup C\left(T_{2}\right)|\cdot |C\left(T_{1}\right)|}\\ \;+\frac{\sum_{a\in C\left(T_{1}\right)\backslash C\left(T_{2}\right)}\sum_{b\in C\left(T_{2}\right)}|a⊕b|}{|C\left(T_{1}\right)\cup C\left(T_{2}\right)|\cdot |C\left(T_{2}\right)|}, \end{array}$$

where a⊕b is the symmetric difference for a pair of sets a and b.

The GRF distance (Equation 2) is calculated by comparing the clusters (or bipartitions) of each tree. For clusters in $T_{1}$ and not in $T_{2}$, the symmetric difference with all clusters in $T_{2}$ is summed. Similarly, for clusters in $T_{2}$ and not in $T_{1}$, the symmetric difference with all clusters in $T_{1}$ is summed. These sums are then normalized by the total number of unique clusters across both trees minus the number of clusters in one of the trees. This normalization ensures that the distance accounts for differences in the sizes of the trees.

Comparing the GRF and RF distances, the main features of the GRF distance can be highlighted as follows. First, by contrast to the RF distance measure, GRF is not limited to trees defined on the same taxa. Second, GRF can be computed in linear time relative to tree size. Third, GRF uses more tree information than the RF distance, due to the fact that GRF considers both shared and non‐shared clades. Finally, the GRF distance, in fact, is not a refined version of the RF distance because they do not always yield equivalent results even for trees with the same set of leaves. The authors state (Llabrés et al., 2021) that for phylogenetic trees on the same set of taxa, $\mathrm{RF}\left(T_{1},T_{2}\right)\leq \mathrm{GRF}\left(T_{1},T_{2}\right)$ and there is no constant C such that $\mathrm{GRF}\left(T_{1},T_{2}\right)\leq C\cdot \mathrm{RF}\left(T_{1},T_{2}\right)$ for all pairs of phylogenetic trees. This indicates that while GRF includes and extends RF by considering additional information (such as non‐shared clades), it does not uniformly refine the RF distance in a straightforward manner. The GRF distance retains the flexibility of the RF distance to be used with both rooted and unrooted trees.

As an example, the GRF distance was calculated between a pair of two trees defined on different but mutually overlapping sets of taxa (trees $T_{1}$ and $T_{2}$ in Figure 1). The result obtained is $\mathrm{GRF}\left(T_{1},T_{2}\right)=2420/551\approx 4.39$.

### 3.4 The dissimilarity measure

The RF distance, as mentioned before, is the most popular distance measure based on splits or bipartitions. Another split‐based distance measure is the dissimilarity measure (Koperwas & Walczak, 2008) that can be used for two trees defined on the same or different leafsets. In order to establish a formula for the dissimilarity measure (DM), two auxiliary definitions are needed.

Definition 1 Let splits $s_{1}$ and $s_{2}$ be restrictively equal on a set of leaves $s_{3}$ if $s_{1}$ and $s_{2}$ are equal after removing leaves that are not part of $s_{3}$.

Definition 2 Let split $s_{2}$ be a supersplit of $s_{1}$ and $s_{1}$ is a subsplit of $s_{2}$ if and only if the set of leaves $s_{1}$ is a subset of the set of leaves $s_{2}$, and $s_{2}$ is restrictively equal to $s_{1}$ on the set of leaves $s_{1}$.

The formula for the dissimilarity measure is as follows:

(3) $$\mathrm{DM}\left(T_{1},T_{2}\right)=1-\frac{|\mathrm{SSF}|}{|S_{1}\tilde{\cup }S_{2}|},$$

where SSF is a strict‐frequent splitset, which is defined as a set comprising subsplits s that are present in all input trees, with the additional condition that there is no other frequent subsplit, also occurring in all input trees, which acts as a supersplit of s. $S_{1}\tilde{\cup }S_{2}$ is the altered sum of both splits, such that if $s_{1}$ is a supersplit of $s_{2}$ for splits $s_{1}\in S_{1}$, $s_{2}\in S_{2}$, then only the supersplit ($s_{1}$) should be included in the result.

The dissimilarity measure based on Formula 3 can be applied to both rooted and unrooted trees, although its emphasis on splits suggests a natural preference for unrooted trees. The main weakness of this measure is that it is not a metric and that it does not consider branch length information.

As an illustration, the two trees from the previous examples are used (see Figure 1). Tree $T_{1}$ includes 10 trivial splits and 7 non‐trivial splits; tree $T_{2}$ contains 15 trivial splits and 12 non‐trivial splits. A split is considered trivial if it contains only one leaf on one side of the split. Non‐trivial splits are shown in Table 1. In this case, $\mathrm{DM}\left(T_{1},T_{2}\right)\approx 0.75$. This value means that the trees $T_{1}$ and $T_{2}$ are dissimilar to a degree of 75%. The dissimilarity measure quantifies how different the trees are, with a value closer to 1 indicating greater dissimilarity. Therefore, a dissimilarity measure of 0.75 implies that the trees are 75% dissimilar, or equivalently, 25% similar.

**TABLE 1:**
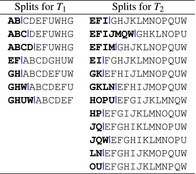
Non‐trivial splits for trees $T_{1}$ and $T_{2}$. In addition to this, $T_{1}$ includes 10 trivial splits, $T_{2}$ contains 15 trivial splits.

*Note*: The trivial splits correspond to the number of species (i.e., leaves or external nodes) present in the tree.

### 3.5 The vectorial tree distance measure

Priel and Tamir (2022) introduced a vectorial tree distance (VTD) measure which is determined as a vector with entries representing the difference in the quantity of branches at each level of the trees. This distance can also be used to compare trees defined on different but mutually overlapping sets of taxa.

In order to describe the approach to calculating the VTD, it is necessary to define a T‐Alignment and a minimal T‐Alignment. A T‐alignment of trees $T_{1}$ and $T_{2}$ is a mapping (denoted as ${\mathrm{AL}}_{T}$) which, for each level (denoted as l) from both roots, maps branches of one tree at that level to branches of another tree at the same level (with the possible addition of zero if the tree has no branches at the examined level) and under the conditions that if branch b from $T_{1}$ T‐aligns with branch $b^{\prime }$ from $T_{2}$, then all descendants of b will be aligned with all descendants of $b^{\prime }$. An ${\mathrm{AL}}_{T}$‐alignment is considered a minimal T‐Alignment if, for each level l, upon iterating over all possible alignments at that level, the difference between the number of descendants of any two T‐aligned branches, as summed across all pairs of T‐aligned branches at that level l, is minimal. Based on this, the VTD of two trees $T_{1}$ and $T_{2}$ is a vector, the l‐element of which is the minimal difference defined by the minimal T‐alignment ${\mathrm{AL}}_{T}$ for level l.

The basic principles of the VTD calculation approach are as follows.

- To begin the calculation of VTD levels, the roots of the trees are identified. The zero element of the distance vector, where numbering begins with zero, is the difference between the number of edges of the tree roots. Specifically, at level 0, the number of edges stemming directly from the root nodes of the two trees is compared. The difference in the number of these edges constitutes the first entry of the distance vector. For example, if one root has three edges and the other root has two edges, the zero element of the distance vector would be 3−2=1.
- The next level (level 1) involves assigning a vector $\left(n_{1},n_{2},\ldots ,n_{k}\right)$ to the roots of both trees, where k is the number of branches in the considered root, and $n_{k}$ is the number of branches of the k‐th branch of the root. If one tree has no branches at the corresponding level relative to another tree, then this vector element (at this level) will be zero. A vector can be defined for any node using this technique. Next, the objective is to find a T‐alignment in which the L1 norm between the two corresponding vectors is minimal. This minimal value is the first element of the distance vector. It is possible that more than one alignment has the same minimal value. In this situation, these alignments are used in the next step (level 2).
- In the level 2, T‐alignments are sought for the next branches based on the minimal T‐alignments identified in the previous step. The node vectors for this level are examined in the same way as the previous level, moving forward accordingly. The second element of the distance vector is the minimum across all sums of L1 norm distances at level 2, passing through all minimum T‐alignments of level 1.
- This procedure is performed recursively for all tree nodes. A dynamic programming method is used to calculate VTD.

There are certain limitations to this approach that need to be considered. First of all, this method ignores all tree labels and branch lengths and, therefore, processes less detailed information. It is crucial to highlight that the resulting distance is not a metric, at least due to the omission of leaf names. Furthermore, the results of this algorithm return a vector, which can be inconvenient to use in some tasks. To address this inconvenience, the authors show that VTD can be represented as a scalar. For instance, for the k‐means algorithm, the authors used the root‐mean‐square of the VTD vector between pairs of trees (Priel & Tamir, 2022). For the k‐medoids algorithm, the sum of distance values starting from level 2 can be calculated. However, it is important to note that the VTD method is designed for rooted trees, as it assumes the presence of a root in each tree, which means it may not be directly applicable to unrooted trees without modifications.

The complexity of the VTD can be estimated as $O\left(|\text{Match}\left(m\right)|\cdot |V|\right)$, where $|\text{Match}\left(m\right)|$ is the number of minimal matching of two m‐dimensional vectors, and ∣V∣ is the maximum number of nodes.

VTD was calculated for the two trees described in the first example (see Figure 1). This involved converting each tree into its corresponding adjacency matrix, identifying their roots, and calculating the vectorial tree distance. The resulting vector is (1, 2, 6, 8). Adding up the elements of this vector starting from level 2 yields a value of 16. The calculation of the root‐mean‐square of the vector returns 4.6.

### 3.6 Geodesic in the extended BHV tree space

A phylogenetic tree can be represented in the Billera‐Holmes‐Vogtmann (BHV) tree space introduced and described by Billera et al. (2001). Trees in BHV are represented by splitting weights, which are the lengths of internal edges associated with the non‐trivial splitting. This space consists of Euclidean subspaces named orthants, where each orthant is represented by phylogenetic trees with different branch lengths but the same topology. Orthants are connected with each other if they share the same splittings in the topology of the tree. However, in low‐dimensional BHV tree space orthants, tree topologies have internal vertices with degrees higher than 3.

The BHV tree space utilizes the geodesic as its internal metric, which is defined as the shortest path between two points that lies entirely within the space, and the geodesic is unique (Billera et al., 2001).

The geodesic length provides the distance between two trees in the BHV tree space (Billera et al., 2001). There are several papers presenting algorithms for calculating the geodesic distance (e.g., Battagliero et al., 2010; Kupczok et al., 2008; Owen & Provan, 2010). Our case considers phylogenetic trees with non‐identical but overlapping sets of taxa, and extending the BHV approach could allow processing such trees. Ren et al. (2017) approach this problem by providing a combinatorial algorithm that expands tree topologies to areas in higher‐dimensional tree spaces. This makes it possible to identify which topologies include a given tree as partial data.

Ren et al. (2017) introduce several structures, such as a BHV connection cluster, a BHV connection space, and a BHV connection graph in their work. These elements are essential components of the approach and can be described as follows. Let $n_{i}$ be the initial number of leaves in tree $T_{1}$, and $n_{l}$ be the number of new leaves to be added to $T_{1}$. A BHV connection cluster is defined as a collection of objects comprising the initial unweighted binary tree $T_{1}$, $n_{i}$, and $n_{l}$. Thus, a BHV cluster contains $n_{i}+n_{l}$ leaves. A BHV connection space is a set of orthants of dimension $n_{i}+n_{l}-3$ and related orthants of lower dimension. Trees in this space are represented as points, and the coordinates of these points are the weights of the tree splits. It should be noted that the BHV connection space has the same set of leaves as the corresponding connection cluster. The dimensionality of the space is specified by the number of leaves of an unrooted tree. A BHV connection graph is a graph with a vertex set consisting of one‐dimensional orthants in the connection space or a set of splits in all trees in the connection cluster.

Based on these structures, Ren et al. (2017) explain the procedure for constructing the BHV connection graph, navigating through multiple instances of the BHV space from lower to higher dimensions. This sophisticated technique is employed to facilitate the precise calculation of distances between phylogenetic trees exhibiting varying numbers of leaves within the BHV tree space. However, the method described for connecting BHV spaces and computing geodesic distances is designed specifically for unrooted trees.

Research in the use of BHV tree space for handling trees with different but overlapping sets of taxa has also been conducted by Grindstaff and Owen (2018), who focus on unrooted trees. Their approach utilizes the tree dimensionality reduction map Ψ, which produces a map from a tree space with dimension $|L_{1}|$, where $L_{1}$ is the size of the leaf set in tree $T_{1}$, to a tree space with dimension $|L_{2}|$, where $L_{2}$ is the size of the leaf set in tree $T_{2}$, such that $|L_{1}|<|L_{2}|$. This lower dimension space includes all trees with a subset of leaves $L_{2}\subset L_{1}$. They create a preliminary image $\Psi^{-1}$ of this map in order to reconstruct information about the initial tree T from images $\Psi_{L_{2}}\left(T\right)$ for the different set $L_{2}$.

Grindstaff and Owen (2018) show how to efficiently calculate $\Psi^{-1}$, which results in all trees with the entire set of leaves that map to the tree T. Their algorithm computes the extension space, which is the set of all phylogenetic trees resulting from adding $n_{l}$ new leaves to T. The authors specify two parameters, α and p, that measure the metric distortion degree for a tree collection $T_{i}$ with combinatorial compatibility constraint, that is, when the connection cluster for these trees is not empty. These parameters indicate the minimum acceptable error in building a supertree from a collection of trees $T_{i}$. These variables are derived through linear optimization problems, which are associated with the equations defining the spaces of approximate solutions, and can be directly calculated using linear programming methods. This framework extends the outcomes and definitions from Ren et al. (2017).

The geodesic in the BHV tree space has several advantages over other distance measures in terms of the tree information they use and process. First, the representation of phylogenetic trees in the BHV tree space utilizes tree topology and branch length, which are very important features of phylogenetic trees. Second, the geodesic is a metric better suited to comparison tasks. Third, researchers initially worked on how to efficiently calculate the geodesic for trees defined on the same taxa, and there are papers (Grindstaff & Owen, 2018; Ren et al., 2017) on extending existing approaches for phylogenetic trees with different numbers of leaves. However, this feature also has its drawbacks, the major one being computational complexity. In fact, creating additional structures, such as a connection cluster, a connection space, and a connection graph, and moving from lower to higher dimensions increases the computational complexity to $O\left(n^{l+2}\right)$, where n is the total number of leaves and l is the number of new leaves to be added to the tree. This is critical for big trees with large l. This is also complemented by the challenges of computing the geodesic distance. Work on extended versions of the BHV tree space, which allow handling phylogenetic trees defined on different but overlapping sets of taxa, is currently more theoretical. The practical implementation of these extended versions is still under development.

### 3.7 Other approaches

In most cases, when dealing with trees defined on different but overlapping taxa sets, leaf imputation can be applied (as shown earlier). The different approaches to tree completion developed by a number of researchers are worth mentioning. Rabiee and Mirarab (2020a) introduced a method called INSTRAL (Insertion of New Species using asTRAL) to add a new species into a species tree, considering the existing collection of gene trees that already include the new species, with the aim of minimizing the overall quartet discordance between the updated tree and the gene trees. Later in 2020, the authors described an algorithm for completing a constraint species tree making use of individual gene trees (Rabiee & Mirarab, 2020b). This method is based on the algorithm developed by Bansal (2018), calculating the LCA (least common ancestor) mapping in both directions and allowing multifurcating of input and output trees. The solution was implemented in the ASTRAL (Accurate Species TRee ALgorithm) program (Zhang et al., 2018) to infer a species tree.

Another approach is based on the completion of gene trees without considering species trees presented by Mai and Mirarab (2022). The task is to complete each gene tree relative to other gene trees in a manner that its total distance to other trees is minimal. The authors extended the B13 algorithm by Brodal et al. (2013), which can enable insertion of new species into a tree under maximization of its total quartet score in relation to a given set of trees. They refined the asymptotic complexity of quartet‐based taxon imputation (for species trees or gene trees) and implemented a quartet subsampling procedure, which improves the accuracy of the completion of gene trees.

Mahbub et al. (2022) address the issue of missing data in phylogenetic datasets by formulating the Quartet Distribution Imputation problem. The authors presented Quartet‐based Gene Tree Imputation using Deep Learning (QT‐GILD), which is a deep learning‐based method that explores the quartet distribution produced by a specified set of incomplete gene trees. The technique utilizes a purpose‐designed autoencoder (AE) model and generates a complete set of quartets. This method assists both in adding missing quartets and in correcting the quartets present in a given set of estimated and incomplete gene trees using an appropriate distribution of quartets.

Yasui et al. (2018) proposed an innovative optimization‐based imputation method that infers missing distances between leaves in gene trees using a mixed integer non‐linear programming model. They introduced a comprehensive pipeline named *imPhy*, which simulates gene trees, imputes the missing pairwise distances, reconstructs the gene trees, and evaluates the efficiency of these reconstructions. The principle of their method involves a two‐stage optimization approach. In the first stage, the method assigns individuals to clades based on available distance data, using an integer non‐linear mathematical program. This program minimizes the p‐norm between the distances of two leaves within the same clade relative to another leaf. In the second stage, it imputes the missing distances by leveraging the assigned clades, solving a mathematical program to estimate the distances based on the available data. This approach ensures that the imputed distances are consistent with the structure of the gene trees.

Yoshida (2023) introduced an approach to imputing missing parts of phylogenetic trees through tropical geometry. This method exploits the properties of tropical polytopes, which are convex hulls defined in the tropical metric space, to improve the accuracy of imputations. This approach consists of several key steps. Initially, induced trees are computed on the observed set of leaves from the training set. These trees are compared, and the tropical polytope is constructed from those with the closest topologies. The tree with missing data is then projected onto this tropical polytope. The use of tropical geometry provides a fitting framework for these imputations. The method ensures that the imputed phylogenetic tree is close to the true tree, with the worst‐case scenario resulting in a Robinson–Foulds distance of four.

In addition, mention should be made of the Tree Edit Distance (TED) measure, which can be defined as the minimum number of removals, inserts and replacements required to transform one tree into another. TED is widely covered in several works (e.g., Akutsu, 2010; Schwarz et al., 2017; Shin, 2015), including practical implementation to phylogenetic trees. In particular, Koperwas and Walczak (2011) presented an approach for TED applied to trees without being necessarily composed of the same set of taxa. The authors introduce contraction and pruning operations with corresponding costs for tree edits, and their TED is defined as the minimum sum of the specified editing costs (contraction and pruning) that can be performed on two trees in order to transform one tree into another. This implementation of TED provides that it is a metric.

There are other approaches, not directly addressing measures or algorithms for calculating distances, but related to the visual comparison of phylogenetic trees defined on different but overlapping sets of taxa. Visual TreeCmp developed by Goluch et al. (2020) and iPhyloC created by Hammoud et al. (2022) tools are worth highlighting due to their ability to analyze shared and non‐shared taxa and to work with trees in the context of supertrees. IPhyloC is an interactive web service allowing side‐by‐side comparison of phylogenetic trees. The tool takes binary and non‐binary trees as input, provides automatic detection of common taxa for trees (highlighting them with color), pruning of trees to common taxa, color changes for some taxa, and additional visualization capabilities.

Visual TreeCmp is a tool for comparing phylogenetic trees with the implementation of a number of existing distance measures, such as the triplet distance, the Robinson–Foulds distance, the geodesic, and the quartet distance. All of these measures are designed to calculate the distance between trees with the same set of taxa, but when the user needs to derive a distance for trees defined on different but overlapping sets of taxa, the tool gives the ability to prune trees to their common taxa and compare them using the pre‐defined measures. Visual TreeCmp uses Phylo.io (Robinson et al., 2016) to visualize some results, and it is worth noting that Phylo.io itself is a good tool for side‐by‐side comparison of phylogenetic trees through their visualization.

## 4 MATERIALS AND METHODS

The distance measures described above are comparable from different points of view. First, the RF‐based distance measures (i.e., RF(−), RF(+), and GRF) can be compared on a dataset of phylogenetic trees defined on different but overlapping sets of taxa. Second, all considered measures are commensurable on the basis of their properties, such as the tree information used (e.g., topology, branch length, leaf names, and bootstrap), tree types (binary or non‐binary), and computational complexity. Finally, it is essential to emphasize and summarize the advantages and disadvantages of the reviewed distance measures.

The procedure for comparing distance measures based on data includes the following steps: (1) generating clusters of phylogenetic trees with different but overlapping taxa, (2) calculating the selected distance measures, and (3) visualizing the results. The GPTree (Koshkarov & Tahiri, 2023a) and GPTree Cluster (Koshkarov & Tahiri, 2023b) phylogenetic tree generators were used to generate the phylogenetic tree dataset. This tool is used to generate clusters of phylogenetic trees with specified parameters, including number of trees, minimum and maximum number of leaves, and level of overlap between trees. Each cluster is based on a species tree, from which the required number of gene trees is generated.

Three datasets of phylogenetic trees with four clusters in each were generated with the following parameters. For all datasets, the number of trees is 5 and the range (minimum and maximum) for the number of leaves is between 14 and 16. The datasets differ among themselves in the level of overlap, which is 50%, 60%, and 70%.

Next, the distances (based on the considered measures) between each tree in datasets are calculated. To calculate RF(−), a technique of pruning both trees to their common set of leaves and computing the classical RF distance on the resulting trees is used (Equation 1). To calculate RF(+), the procedure of adding non‐common leaves of one tree to another, described in the Related work section, is utilized. The resulting trees have the same set of taxa and the formula for calculating the RF distance is applied to these trees. GRF is calculated based on Equation 2. In total, nine cases (three distance measures per three levels of overlap) can be analyzed.

**FIGURE 3:**
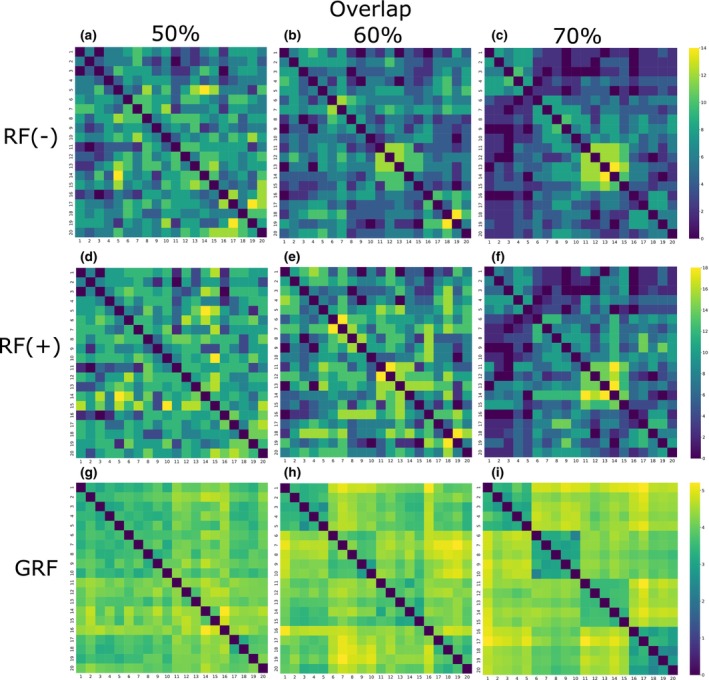
Heatmap visualization of RF(−), RF(+), and GRF values relative to 50%, 60%, and 70% overlap levels. The datasets comprise four clusters of five phylogenetic trees each. Each of the nine plots represents the calculated values of the indicated distance measures. Three plots are presented for each level of overlap. The RF(−) values in plots (a–c) and the RF(+) values in plots (d–f) demonstrate no clear separation of the trees by color hue. However, the GRF for overlap levels of 60% (h) and 70% in plot (i) show a clearer separation of clusters from each other. In addition, RF(−) and RF(+) show zero distance values for non‐identical trees.

**FIGURE 4:**
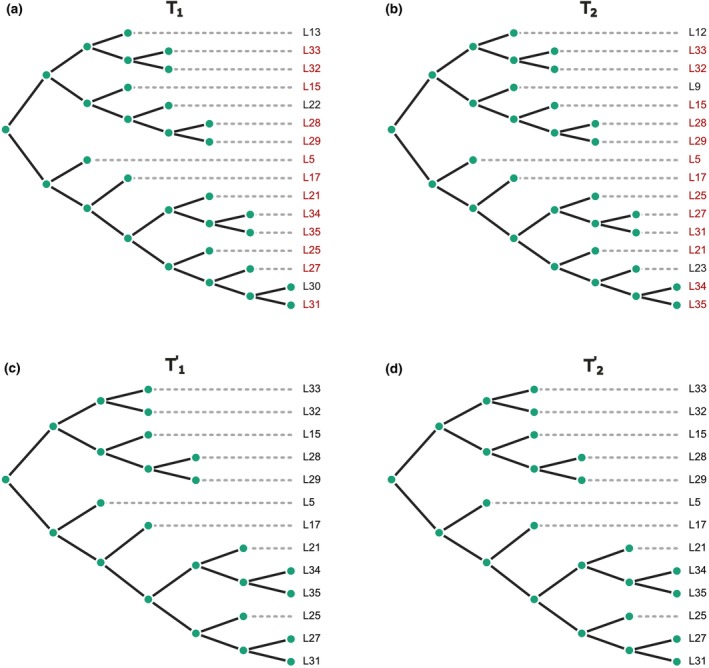
Illustration of tree completion where RF(−) equals zero for non‐identical trees: Trees (a) $T_{1}$ and (b) $T_{2}$ are non‐identical but share the same topology through common leaves (highlighted in red). Subsequently, the resulting trees, (c) $T_{1}^{\prime }$ and (d) $T_{2}^{\prime }$ became identical in topology by pruning all leaves except the common leaves.

**FIGURE 5:**
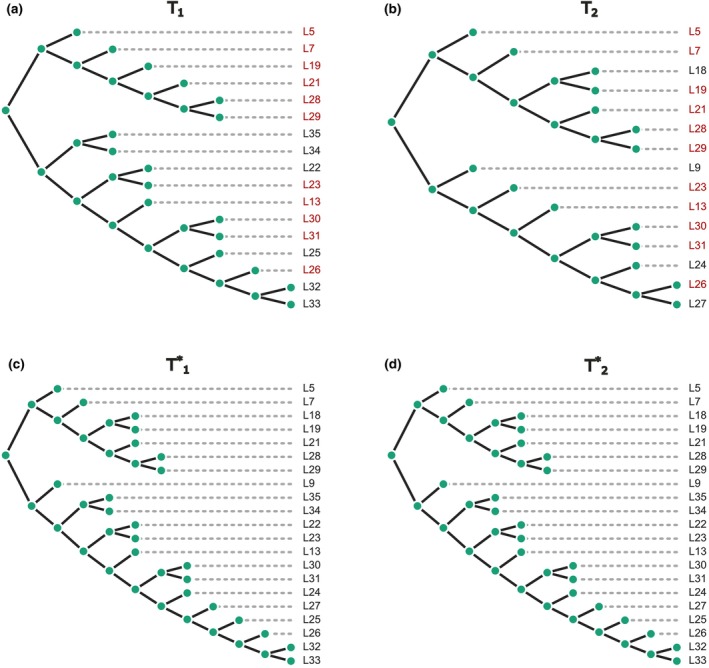
Illustration of tree completion where RF(+) equals zero for non‐identical trees: Trees (a) $T_{1}$ and (b) $T_{2}$ are non‐identical but share the same topology through common leaves (highlighted in red). Subsequently, the resulting trees, (c) $T_{1}^{*}$ and (d) $T_{2}^{*}$, achieve topological identity by seamlessly integrating unique leaves from one tree into the other. RF(+) strategically optimizes the placement of new leaves, effectively minimizing the Robinson–Foulds (RF) distance to zero—the absolute minimum achievable.

The next step is to calculate all pairwise distances between all generated datasets and construct a heat map associated with the overlap levels (50%, 60%, and 70%) and considered distance measures (RF(−), RF(+), and GRF), as shown in Figure 3. This heat map helps visualize the relationships and potential separations among clusters based on the different distances. In addition, this approach may reveal cases where two non‐identical phylogenetic trees have a distance equal to 0 after applying the chosen distance method. If such a situation exists, a more detailed comparison and visualization of the corresponding pairs of trees will be performed (see Figures 4 and 5).

**FIGURE 6:**
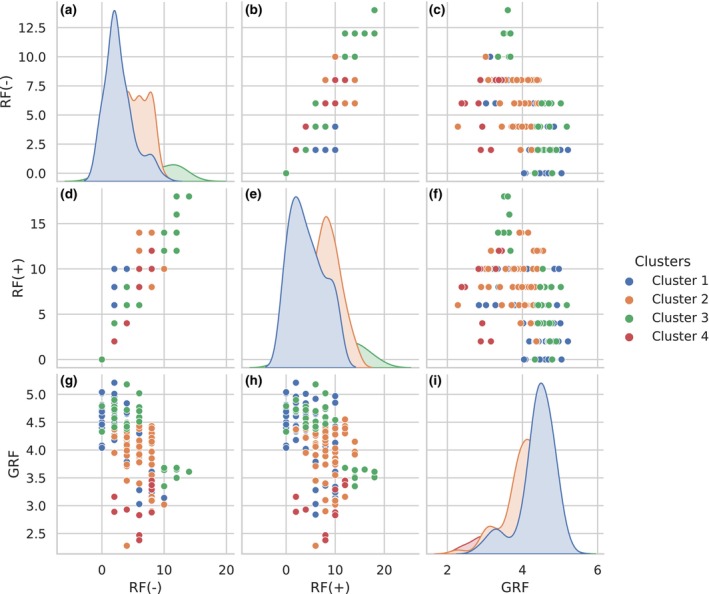
Comparison of distance measures RF(−), RF(+), and GRF on the generated dataset. The calculation includes a pairwise comparison of phylogenetic trees. The plots (a, e, and i) on the diagonal show the distribution of the corresponding measures across clusters. The remaining graphs show the possible relationship between the values in terms of correlation. The X‐axis includes the calculated values of RF(−), RF(+), and GRF, while the Y‐axis encompasses the values of GRF, RF(+), and RF(−). A strong correlation between RF(−) and RF(+) in plots (b and d) and the absence of a significant relationship between GRF and the other two distances in plots (c, f, g, and h) can be detected.

The visualization of the results makes it possible to compare the distribution of values for the considered distance measures. In order to observe these values and identify potential correlations between the distance measures under consideration, the results for a dataset with the overlap level that shows the best cluster separation from the previous analysis will be visualized (see Figure 6). The Pearson's correlation is used to assess the relationship between the considered distance measures, since it provides a clear, standardized, and interpretable indicator for the linear association between these measures.

For comparison purposes, information regarding whether the considered measure is a metric or not will be highlighted. To be a metric, a distance measure d must satisfy the following properties for any three phylogenetic trees $T_{1}$, $T_{2}$, and $T_{3}$:

1. $d\left(T_{1},T_{2}\right)\geq 0$ (non‐negativity),
2. $d\left(T_{1},T_{2}\right)=0$ if and only if $T_{1}=T_{2}$ (identity of indiscernibles),
3. $d\left(T_{1},T_{2}\right)=d\left(T_{2},T_{1}\right)$ (symmetry),
4. $d\left(T_{1},T_{3}\right)\leq d\left(T_{1},T_{2}\right)+d\left(T_{2},T_{3}\right)$ (triangle inequality).

## 5 RESULTS AND DISCUSSION

Given the prevalence and widespread acceptance of the classical Robinson–Foulds distance in the field of phylogenetics for the comparison of phylogenetic trees, it is judicious to scrutinize RF‐based distances that have been adapted for trees defined on different but mutually overlapping sets of leaves.

For the generated dataset containing 20 phylogenetic trees in 4 clusters with the parameters defined above, the RF(−), RF(+), and GRF values were calculated for 50%, 60%, and 70% overlap levels for each pair of trees. The results for the nine cases (three distance measures per three levels of overlap) are presented in Figure 3. This analysis enables the identification of specific peculiarities.

First, for an overlap level of 50% (and actually less), there is no clear pattern of separation of clusters from each other (every subsequent five trees form one cluster in the data). The RF(−) and RF(+) distances also do not clearly distinguish clusters for 60% and 70% overlap levels. The GRF distance for 60% overlap and further for 70% overlap shows clearer separation highlighted by color. Each of the four tree clusters (Figure 3i) has a darker hue, which corresponds to lower values of distance between trees in the cluster, and a brighter hue, which indicates higher values of distance between trees from different clusters. This may show a better ability of GRF to identify clusters in data (trees within a cluster are closer to each other and more distant from trees in another cluster) and to use it in the tasks of clustering phylogenetic trees defined on different but overlapping taxa. In addition, using GRF distance does not require tree manipulation unlike RF(−) and RF(+).

Second, RF(−) and RF(+) for all three cases (50%, 60%, and 70% overlap) have multiple zero values not on the main diagonal (in fact, the zeros on the main diagonal show the distance between trees with themselves). This means that there are non‐identical trees between which the distances RF(−) and RF(+) can be zero. Moreover, the greater the level of overlap, the more zero values can be observed.

The case of two non‐identical trees that have RF(−) distance equal to zero (after a tree pruning operation) is shown in Figure 4. The process of pruning two trees to their common taxa can also result in the removal of useful information about these trees. The RF(−) distance can take zero values for two non‐identical trees if these trees have the same topology for their common leaves. Obviously, in this case the trees become identical after pruning.

The case of two non‐identical trees that have RF(+) distance equal to zero (after tree completion) is shown in Figure 5. This case is less trivial because the completion procedure is performed for both trees. First, this example also shows that the topology for common leaves is the same. Second, the non‐common leaves of one tree are added to the other tree while minimizing the RF distance, and this optimal completion can make the trees identical in terms of their topology, where the minimum RF value is zero.

Figure 6 shows the distribution of all three distance values for RF(−) (Figure 6a–c), for RF(+) (Figure 6d–f), and GRF (Figure 6g–i) for each pair of tree with the overlap level that shows the best cluster separation from the previous result (70%). These distributions for all four clusters indicate that the maximum values for each distance measure retain their order (clusters 1, 2, 3, and 4, respectively). In addition, a strong positive correlation (Pearson's correlation coefficient is 0.9) between RF(−) and RF(+) can be observed (Figure 6b,d).

The distance measures under review can also be compared based on their properties, such as the tree information used (topology, bootstrap, branch lengths, and leaf names) and their computational complexity. This comparison is shown in Table 2. For RF(−) and RF(+), the metric properties of the classic RF distance are used. The RF(−), RF(+), GRF, and geodesic (BHV) distances are all metrics. They satisfy the four required properties, making them reliable measures for various phylogenetic analyses. The DM and VTD measures, however, are not metrics. DM does not satisfy the triangle inequality, which can lead to inconsistencies in measuring distances between trees. VTD does not satisfy metric properties due to its disregard for leaf names.

**TABLE 2:** Comparison of distance measures for trees with non‐identical taxa: Evaluating metric properties and associated parameters.

Methods	Metric properties	Complexity	Non‐binary trees	Branch length	Topology	Rooted trees	Unrooted trees	Leaf names	Bootstrap	Reference
RF(−)	Yes	$O\left(n\right)$	No	No	Yes	Yes	Yes	Yes	No	Bansal (2020), Cotton and Wilkinson (2007)
RF(+)	Yes	$O\left(n_{1}k^{2}\right)$	No	No	Yes	Yes	Yes	Yes	No	Bansal (2020), Yao and Bansal (2021)
GRF	Yes	$O\left(n\right)$	No	No	Yes	Yes	Yes	Yes	No	Llabrés et al. (2021)
DM	No	$O\left(n^{2}\right)$	No	No	Yes	Yes	Yes	Yes	No	Koperwas and Walczak (2008)
VTD	No	$O\left(|M\left(m\right)|\cdot n_{2}\right)$	Yes	No	Yes	Yes	No	No	No	Priel and Tamir (2022)
BHV	Yes	$O\left(n^{l+2}\right)$	No	Yes	Yes	No	Yes	Yes	No	Grindstaff and Owen (2018), Ren et al. (2017)

*Note*: A distance measure is considered a metric if it satisfies the four properties of non‐negativity, identity of indiscernibles, symmetry, and the triangle inequality. In this context, n denotes the total number of unique leaves in both trees, $n_{1}$ represents the total number of nodes in the tree, k reflects the quantity of maximal subtrees unique to an input tree, $|M\left(m\right)|$ indicates the number of minimal matchings of two m‐dimensional vectors, $n_{2}$ signifies the maximum number of nodes, and l represents the number of leaves to be added to the tree.

While bootstrapping has not been incorporated into any of the methods listed in Table 2, it is important to consider its potential relevance in future research. Bootstrapping can provide additional information about the reliability of the inferred phylogenetic relationships, which could be integrated into distance measures to handle more comprehensive information about the phylogenetic trees being compared. Including bootstrap values in distance measures could enhance the robustness of tree comparisons by weighting the importance of clades based on their bootstrap support. For example, trees with highly supported clades (high bootstrap values) could be given more significance in the comparison process, potentially leading to more accurate and reliable distance calculations.

The main advantages and drawbacks of the reviewed distance measures are shown in Table 3.

**TABLE 3:** Analysis of distance measures for phylogenetic trees with non‐identical but overlapping sets of taxa: Uncovering strengths and limitations in tree‐based analyses.

Methods	Main strengths	Main limitations
RF(−)	Easy to understand and computationally efficient. Applicable to trees of varying sizes, including large trees with a large number of taxa	Can lead to the loss of valuable topological information by discarding leaves not shared by both trees. Distance can be zero for non‐identical initial trees. Also incorporates basic shortcomings of the classic RF distance
RF(+)	Effectively considers all topological information from both compared trees, using a wider range of possible values. Makes better use of available topological information	Possesses the main drawbacks of the classic RF distance. Time‐consuming if the completion process involves trees with a large number of non‐common leaves. Distance can be zero for non‐identical initial trees
GRF	Intuitive, with a natural interpretation in terms of common splits and processes shared and non‐shared taxa. A metric that can be computed in linear time	Does not exploit branch length information
DM	Easy to compute, showing how many subsplits (ratio) the considered trees share in common	Not a metric and does not consider branch length information
VTD	Efficiently distinguishes symmetric trees from asymmetric trees, and hierarchical trees from non‐hierarchical trees	Ignores tree leaves and branch lengths, processing weaker information
BHV	Uses broader information about the trees under comparison, including tree topology and branch lengths. A metric. The geodesic distance considers the curved nature of the BHV space, providing a more accurate representation of true distances between trees	Computation is time‐consuming, especially when many leaves have to be added to the trees being compared. BHV tree space is a complex, non‐Euclidean space with curvature, making the computation of the geodesic distance challenging

Given the comprehensive overview of several distance measures for comparing phylogenetic trees defined on different but overlapping sets of taxa, the choice of a distance measure should be guided by the specific needs of the task, considering the strengths and limitations of each method. Here are some guidelines for choosing a distance measure in a particular context.

In situations where a rapid comparison is required, with a particular focus on topology over branch lengths, the RF(−) method is the recommended approach due to its computational efficiency and simplicity. This method is particularly useful for large trees, but should be used with caution as it may discard valuable topological information after the tree pruning procedure. RF(−) can be applied to both rooted and unrooted trees.

In instances where the incorporation of extended topological information is crucial, the RF(+) method, despite its time‐consuming nature, effectively incorporates all topological data from both trees. It is especially beneficial when the complete representation of trees is required for the analysis. However, its computational demand increases with the number of non‐common leaves. RF(+) is suitable for both rooted and unrooted trees.

For a balanced approach that includes shared and non‐shared taxa without considering branch lengths, the GRF method offers an intuitive metric that can be computed efficiently. It is suitable for analyses where common splits and differences in taxa distributions are of interest. GRF can be applied to both rooted and unrooted trees and uses topological information only.

The DM approach can be applied to both rooted and unrooted trees and uses topological information only. This measure is useful for analyses focusing on the structural similarities and differences between trees without considering branch lengths.

The VTD method is a recommended approach when analyzing tree symmetry and hierarchy without focusing on leaf identity or branch lengths. Its efficiency in distinguishing tree structures (even for non‐binary trees) makes it suitable for specific structural analyses, though it processes weaker information due to its disregard for leaf names and branch lengths. VTD is designed for rooted trees.

For comprehensive analyses that incorporate both topology and branch lengths, the extended BHV method is a suitable choice despite its computational complexity. It is particularly appropriate for detailed studies where an accurate representation of distances is critical. This method is recommended for research with the capability to manage the computational demand, especially when comparing trees with many non‐common leaves. The extended BHV method is suitable for unrooted trees.

Metrics like RF(−), RF(+), GRF, and BHV offer robust and consistent measures, while non‐metric measures like DM and VTD provide valuable, albeit less consistent, insights depending on the analysis context.

In summary, the selection of a distance measure for phylogenetic tree comparison should be informed by the specific research objectives, the nature of the trees being compared (e.g., size, binary or non‐binary, importance of branch lengths, rooted or unrooted), and the computational resources available. Each method has its unique strengths and limitations, making it essential to align the choice of the distance measure with the goals of the analysis.

## 6 CONCLUSION

We have reviewed the state‐of‐the‐art approaches to comparing phylogenetic trees with different but mutually overlapping sets of taxa. Methods that allow calculating distances between trees with such properties play an essential role in phylogeny due to a number of tasks requiring comparison of these trees, including supertree construction, phylogeny clustering, and phylogenetic database searches. Among the existing solutions, researchers can choose the measures that meet their needs, for example, using only tree topology (as in RF‐based approaches) or using an extended arsenal of features, including branch lengths. In most cases, it is also important to consider the computational complexity of the methods used, especially in scenarios of big trees with a large number of non‐common leaves.

RF(−) is recommended for use with large trees in order to facilitate rapid, topology‐focused comparisons, which necessitate the use of efficient computation. For detailed topological analysis, RF(+) should be used when a complete representation of trees is needed, GRF is suitable for an efficient, balanced approach considering shared and non‐shared taxa, and DM is appropriate for structural analyses focusing on topological differences. For comprehensive analyses incorporating branch lengths, the extended BHV method is the preferred choice for accurate distance representations when computational resources are available. For specific structural analyses focusing on symmetry and hierarchy, VTD is recommended for rooted trees without considering branch lengths and leaf names.

At present, no methods have yet been developed that can utilize tree topology, branch lengths, bootstrap, and leaf names in a single metric with polynomial time complexity for processing phylogenetic trees with different but overlapping taxa. Under these circumstances, there are possibilities for extending the capabilities of existing methods. For example, the ideas of the RF(+) approach, which uses RF distance as the basic measure to be minimized, can be applied to another distance measure to complete both trees so that they become defined on the same taxa. Similarly, novel techniques can be introduced in the BHV tree space to handle trees with different but overlapping taxa more efficiently, with less complexity. Finally, new approaches to calculating the distance between trees defined on different but overlapping taxa, considering, among other things, branch lengths, can be developed.

## AUTHOR CONTRIBUTIONS

**Wanlin Li:** Conceptualization (equal); data curation (equal); formal analysis (equal); validation (equal); writing – original draft (equal). **Aleksandr Koshkarov:** Conceptualization (equal); data curation (lead); investigation (equal); methodology (equal); validation (equal); visualization (equal); writing – original draft (equal); writing – review and editing (equal). **Nadia Tahiri:** Conceptualization (equal); funding acquisition (lead); investigation (equal); methodology (equal); resources (lead); supervision (lead); validation (equal); writing – review and editing (equal).

## FUNDING INFORMATION

This work was supported by the Natural Sciences and Engineering Research Council of Canada (A.K.: CGS‐D ‐ 589644, and N.T.: RGPIN‐2022‐04322), the University of Sherbrooke grant, and the Fonds de recherche du Québec – Nature et technologies (N.T.: 326911).

## CONFLICT OF INTEREST STATEMENT

The authors declare no conflict of interest.

## Data Availability

The datasets generated and analyzed during the current study are available here: https://github.com/tahiri‐lab/GPTree/tree/GPTreeCluster/datasets.
